# Liposomal Epigallocatechin-3-Gallate for the Treatment of Intestinal Dysbiosis in Children with Autism Spectrum Disorder: A Comprehensive Review

**DOI:** 10.3390/nu15143265

**Published:** 2023-07-24

**Authors:** Jose Enrique de la Rubia Ortí, Costanza Moneti, Pilar Serrano-Ballesteros, Gloria Castellano, Raquel Bayona-Babiloni, Ana Belén Carriquí-Suárez, María Motos-Muñoz, Belén Proaño, María Benlloch

**Affiliations:** 1 Department of Basic Medical Sciences, Catholic University of Valencia San Vicente Mártir, 46001 Valencia, Spain; raquelbayona03@mail.ucv.es (R.B.-B.); beprool@mail.ucv.es (B.P.); maria.benlloch@ucv.es (M.B.); 2 Doctoral School, Catholic University of Valencia San Vicente Mártir, 46001 Valencia, Spain; costanza.moneti@mail.ucv.es; 3 Research Department of Sesderma Laboratories, 46001 Valencia, Spain; p.serrano@sesderma.com; 4 Centro de Investigación Traslacional San Alberto Magno (CITSAM), Catholic University of Valencia San Vicente Mártir, 46001 Valencia, Spain; gloria.castellano@ucv.es; 5 Department of Personality Psychology, Treatment and Methodology, Catholic University of Valencia San Vicente Mártir, 46100 Valencia, Spain; maria.motos@ucv.es; 6 Child Neurorehabilitation Unit, Manises Hospital, 46940 Valencia, Spain

**Keywords:** Autism Spectrum Disorder (ASD), microbiota, epigallocatechin-3-gallate (EGCG), inflammation, oxidative stress

## Abstract

Autism Spectrum Disorder (ASD) is characterized by varying degrees of difficulty in social interaction and communication. These deficits are often associated with gastrointestinal symptoms, indicating alterations in both intestinal microbiota composition and metabolic activities. The intestinal microbiota influences the function and development of the nervous system. In individuals with ASD, there is an increase in bacterial genera such as *Clostridium*, as well as species involved in the synthesis of branched-chain amino acids (BCAA) like *Prevotella copri*. Conversely, decreased amounts of *Akkermansia muciniphila* and *Bifidobacterium* spp. are observed. Epigallocatechin-3-gallate (EGCG) is one of the polyphenols with the greatest beneficial activity on microbial growth, and its consumption is associated with reduced psychological distress. Therefore, the objective of this review is to analyze how EGCG and its metabolites can improve the microbial dysbiosis present in ASD and its impact on the pathology. The analysis reveals that EGCG inhibits the growth of pathogenic bacteria like *Clostridium perfringens* and *Clostridium difficile*. Moreover, it increases the abundance of *Bifidobacterium* spp. and *Akkermansia* spp. As a result, EGCG demonstrates efficacy in increasing the production of metabolites involved in maintaining epithelial integrity and improving brain function. This identifies EGCG as highly promising for complementary treatment in ASD.

## 1. Introduction

Autism Spectrum Disorder (ASD) is a highly heterogeneous and complex disorder characterized by two major groups of core symptoms: (1) persistent deficits in social communication and interaction and (2) restricted and repetitive patterns of behavior, interests, or activities [1,2,3,4,5]. Current epidemiology statistics indicate that the disorder affects approximately 1 in every 160 children worldwide [6].

Characteristic deficits are often associated with a range of gastrointestinal symptoms such as abdominal pain, diarrhea, or constipation [7,8]. Several studies have found that both the composition of the intestinal microbiota and metabolic activities may be altered in individuals with the disorder ASD [9,10]. Therefore, several authors concur that disruptions in the microbiota and intestinal microbiome, i.e., the collection of microorganisms present in the human gastrointestinal tract and their respective genomes [3,11], could trigger many of the gastrointestinal issues experienced by children with ASD, as well as exacerbate some of the core symptoms of the disorder [12,13].

Microbial colonization begins in infancy via the acquisition of maternal microbiota during vaginal delivery [3]. Subsequently, beneficial microorganisms feed on breast milk, which has a high content of oligosaccharides [14]. Similarly, the composition of the microbiota in the early years may be subject to alterations influenced by the delivery method, hygiene habits, and feeding practices and routines. Among these factors, formula feeding has a particularly significant impact [3].

It has been shown that the intestinal microbiota affects the function and development of the immune, metabolic, and nervous systems. Regarding the immune and metabolic systems, intestinal microbiota and its metabolites impact host physiology by regulating the function of the intestinal barrier, redox and mitochondrial metabolism, and mucosal inflammatory response via the regulation of intestinal lymphocytes that provide resistance against potential pathogens [15,16,17,18,19,20]. On the other hand, intestinal microbiota can influence neurochemistry, function, gene expression, and the development of the central nervous system (CNS) through the gut–brain axis, which represents a bidirectional link between the cognitive and emotional functions of the CNS and peripheral intestinal function [15,21,22,23]. It is worth noting that the genus *Bifidobacterium* can metabolize gamma-aminobutyric acid (GABA), *Lactobacillus* spp. can metabolize acetylcholine, *Bacillus* spp. and *Serratia* spp., dopamine, and *Escherichia* spp. and *Saccharomyces* spp., noradrenaline. All these neurotransmitters are essential for the proper functioning of the nervous system as they can enter circulation and directly affect neural processes throughout the body, including the brain [3,24]. Additionally, intestinal proinflammatory cytokines, particularly TNF and IL-6, can have an impact on the brain because they make the blood–brain barrier (BBB) more permeable, allowing peripheral immune cells to enter the brain and stimulating brain cells to produce additional proinflammatory mediators [25,26].

All of this has led to the hypothesis that the development of symptoms related to ASD may be influenced by the disturbance of the gut–brain–microbiota axis caused by changes in the intestinal microbiota.

## 2. Alterations in the Intestinal Microbiota in ASD

The Firmicutes (40–60%) and Bacteroidetes (20–40%) are the two major phyla of bacteria in the healthy human intestinal flora, followed by Proteobacteria, Actinomycetes, *Clostridium* spp., and Verruciformis [3,27]. However, compared to a healthy population, increased levels of bacterial genera such as *Clostridium*, *Desulfovibrio*, and *Ruminococcus* have been observed [15,28], in ASD, along with species that synthesize BCAA such as *Bacteroides vulgatus* and *Prevotella copri*. On the other hand, lower quantities of *Bacteroides fragilis*, *Akkermansia muciniphila*, *Bifidobacterium* spp., and *Enterococcus* spp. Have been found [29,30]. Additionally, the ratio of the *Escherichia/Shigella* genera is altered, as *Shigella* spp. is present at higher levels, while *Escherichia coli* is decreased [31]. 

It should be noted that, despite the consensus regarding these alterations, it is important to consider that not all studies quantifying the microbiota in ASD analyze the same species or genera, leading to some heterogeneity in the results, and various studies emphasize the relationship between the composition of the human intestinal microbiota and the individual’s diet, which can lead to variations in composition depending on the region where the analyses were conducted [32]. In fact, a study in the United States highlighted that children with ASD had lower levels of *Bifidobacterium* spp. And *Prevotella* spp., but higher levels of *Lactobacillus* spp., compared to healthy children [33]. Another study in China described that children with ASD had higher levels of the Actinobacteria and Proteobacteria phyla than the control group [34].

Furthermore, the review conducted by Ho et al. (2020) concluded that although the results of different studies are not entirely consistent [35], some articles indicate that individuals with ASD exhibit a decrease in the percentage of the phylum Bacteroidetes [10,35] or no significant difference [31,36,37] compared to the control group of healthy individuals. This could be due to the fact that within the phylum Bacteroidetes, there are bacteria of the genus *Bacteroides* spp. that are increased, such as *B. vulgatus*, while others are decreased, such as *B. fragilis*, potentially leading to a compensatory effect that results in an unclear profile [30].

The genus Prevotella presents a more variable pattern. In the aforementioned review, the authors noted a lower relative abundance of *Prevotella* spp. in children diagnosed with ASD compared to the control group. However, multiple studies indicate an elevated prevalence of *Prevotella* spp. in children with ASD [15,30,31,38], and this variability could be due to dietary factors. Filippo et al. (2010) compared the composition of *Prevotella* spp. in the microbiota of children following European and African diets and observed a significant increase in *Prevotella* spp. In the microbiota of African children, likely due to their consumption of a grain-rich diet [39]. 

Finally, to establish a correlation between gastrointestinal issues and behavioral problems with the microbiota, the high growth rates of *Clostridium histolyticum*, *Clostridium difficile*, *Clostridium perfringens*, and *Sutterella* spp., the alteration in the *Escherichia/Shigella* genera ratio, and the decreased Bacteroidetes/Firmicutes phylum ratio have been identified as factors associated with gastrointestinal problems. Furthermore, the relative abundance of *Desulfovibrio* spp., *Clostridium* spp., and *Bacteroides vulgatus* has been linked to behavioral disorders [40].

## 3. Polyphenols as a Therapeutic Alternative to ASD: The Epigallocatechin-3-Gallate (EGCG) Option

ASD lacks a medical cure, and understanding molecular pathogenic mechanisms is crucial for proposing alternative therapies to current medications used for treating autism-related symptoms [41]. Given disruptions in the microbiota, exploring the microbiota-gut-brain axis is essential to modify disease development. Polyphenols, as bioactive dietary compounds, show great promise in this regard. These compounds act in the intestine, reducing inflammation and modulating the microbiota and its metabolites. They also have neuroprotective effects on the brain by crossing the BBB [42].

Polyphenolic antioxidants, capable of targeting the intestine and modulating the intestinal microbiota while reducing intestinal inflammation, have been shown to influence memory, cognition, mood, and behavior, thereby contributing to the prevention and treatment of various brain disorders [25]. Polyphenols have garnered attention for their ability to stimulate certain gut bacteria involved in phenolic compound utilization and metabolism, making them a proposed treatment option for neurological disorders [43,44]. Polyphenols are the most ubiquitous phytochemicals in the human diet, with a total daily intake of approximately 1 g [45]. These phenolic compounds, as secondary metabolites in plants, primarily protect them against various aggressions or infections caused by bacteria, insects, or viruses [43,44]. These protective properties in plants are expected to be beneficial for humans as well. Polyphenolic compounds found in green and black tea are the most potent inhibitors of microbial growth. 

These compounds include epigallocatechin-3-gallate (EGCG), epicatechin gallate, epigallocatechin, catechin gallate, epicatechin, and catechin [46]. They have shown significant efficacy in inhibiting the growth of pathogens such as *Helicobacter pylori* [47], *Staphylococcus aureus*, *Escherichia coli O157:H7* [48,49], *Salmonella typhimurium DT104*, *Listeria monocytogenes*, methicillin-resistant *Staphylococcus aureus* [50,51] and *Pseudomonas aeruginosa* [52]. They have also been effective against viruses like HIV and Epstein–Barr [53,54]. 

Among all the polyphenols, several studies highlighted by Duda-Chodak et al. (2015) emphasize the significant role of EGCG [55]. EGCG is the primary catechin in green tea, but it is also found in lower concentrations in certain plant-based foods, such as apples, peaches, kiwis, blackberries, pears, and nuts like pistachios, hazelnuts, and walnuts [56]. 

Hence, considering the advantageous effects observed on the microbiota from polyphenols, including EGCG, a comprehensive mini-review was proposed. This review was based on a thorough scientific search of published studies in PubMed, without any limitations on publication date. The objective was to examine the potential advantages that EGCG might offer to individuals with ASD. The search was from January to April 2023 and utilized MeSH descriptors like “epigallocatechin gallate”, “autism spectrum disorder”, “metabolism”, “microbiota”, and “fatty acids” combined using the boolean operator “AND.”.

### 3.1. Possible Role of EGCG in the Intestinal Microbiota of Patients with ASD

To date, there is a lack of published studies that have used EGCG in ASD. However, there are cellular and animal studies [57,58] that clearly demonstrate the potential of this antioxidant for the clinical treatment of ASD. Additionally, in recent years, several studies have been published employing other polyphenols that are structurally similar to EGCG, such as luteolin or quercetin, which have achieved evident clinical benefits in the disease [59,60,61,62]. Its consumption is linked to reduced psychological distress [63], possibly by inhibiting the growth of specific pathogenic bacteria such as *Clostridium perfringens*, *Clostridium difficile* [64], *Bacteroides ovatus*, and *enterobacteria* (*Salmonella* spp., *Escherichia coli*, *Yersinia pestis*, *Klebsiella* spp., *Shigella* spp., and *Eggerthella* spp.) [49,65,66,67]. Furthermore, in the intestinal microbiota, EGCG increases the abundance of *Bifidobacterium* spp., determined via in vitro studies [65] or in Drosophila models of Parkinson’s disease [66]. EGCG also increases in vitro bacteria of the Bacteroides genus (*Bacteroides uniformis*, *Bacteroides stercoris*, *Bacteroides thetaiotaomicron*, and *Bacteroides cellulosilyticus*) and *Lachnoclostridium* spp. [65] in Male C57BL/6N mice *Akkermansia* spp. [67] and in ovariectomized (OVX) mice fed a high-fat diet (HFD) *Prevotella* spp. [68]. 

A substantial portion of the vital microorganisms in the fecal microbiota of breastfed infants consists of species from the Bifidobacterium genus [69]. *Bifidobacterium* spp. has psychobiotic effects that help reduce anxiety, stress, and other depressive behaviors, particularly strains *B. longum* and *B. breve* [70,71]. This bacterial genus is one of the initial colonizers of the newborn’s intestines, and an imbalance in *Bifidobacterium* spp. can affect infant neurodevelopment [71]. Administering EGCG to increase *Bifidobacterium* spp. has been found to enhance the intestinal microbiota, as supported by previous animal studies that showed an elevated abundance of *Bifidobacterium* spp. in response to a diet enriched with EGCG [72]. 

Bacteria within the Bacteroides genus play a crucial role in breaking down complex molecules, particularly carbohydrates, and confer advantages to their hosts by impeding the colonization of potential pathogens in the digestive tract [73,74]. It has been seen that there are bacteria capable of modulating the immune system. For instance, *B. fragilis* has been shown to restore the balance of T-cell populations in mice affected by ASD [75], while *B. uniformis* enhances the production of anti-inflammatory cytokines and improve metabolic and immune dysfunction [65]. Moreover, this genus can improve social behaviors and physiological abnormalities in individuals with ASD [76], indicating that an increase in *Bacteroides* abundance caused by EGCG could enhance the quality of life in patients. Additionally, this polyphenol can reduce *B. ovatus*, the main intestinal commensal responsible for a systemic antibody response in inflammatory bowel disease [77]. These findings suggest that even within the same genus, EGCG may exert different effects on bacterial species.

*Prevotella* spp. are bacteria that exist in the human microbiota, and are responsible for degrading plant polysaccharides and participating in the synthesis of vitamin B1 [78]. One specific species within the Prevotella genus is *Prevotella copri*, which has been linked to enhanced glucose and insulin tolerance. It is commonly found in individuals who consume a diet rich in fiber, indicating a strong association between the effects of this bacterium and dietary habits. Within the Prevotella genus, *Prevotella copri* can be found. This species has been associated with improved glucose and insulin tolerance and is commonly found in individuals who follow a fiber-rich diet, suggesting a strong connection between the effects of this bacterium and dietary patterns [79,80,81,82]. Consequently, both the bacteria within the *Prevotella genus* and their metabolic activity are significantly relevant to ASD. On one hand, children with ASD present a higher risk of obesity, which implies insulin resistance [83], and, on the other hand, these children exhibit selective eating patterns, with a notable preference for high amounts of simple carbohydrates and low intake of fiber [84]. This dietary behavior is associated with lower levels of *Prevotella* spp. within the microbiota. Additionally, it is worth noting that individuals with ASD have been reported to have low levels of vitamin B1, which plays a significant role in antioxidant activity [85]. 

In relation to the Lachnoclostridium genus, it has been seen to possess anti-inflammatory properties and contribute to the maintenance of intestinal balance by producing butyric acid [86]. This metabolite aids in the elimination of intestinal gases, and its absence is associated with irritable bowel syndrome (IBS) [87]. Therefore, the increase in *Lachnoclostridium* spp. mediated by EGCG [56] can be seen as having a positive impact on the microbiota of ASD patients.

EGCG modulates intestinal microbiota and its metabolites, enriching the population of short-chain fatty acid (SCFA)-producing bacteria such as *Akkermansia* spp., resulting in an improvement in the production of acetate, propionate, and butyrate in a sodium dextran sulfate (SDS)-induced colitis mouse model, where levels of these metabolites were significantly reduced [88,89]; this promotes an anti-inflammatory and antioxidant state in the intestine. [88]. *Akkermansia* spp. utilizes intestinal epithelial mucin as a source of energy. By degrading mucin, it releases nutrients such as monosaccharides, amino acids, and SCFAs, which are used by other bacteria in the microbiota, stimulating their metabolic functions [90]. *Akkermansia* spp. is involved in the regulation of glucose metabolism and adipose tissue homeostasis. Therefore, the fact that EGCG increases the levels of *Akkermansia muciniphila* improves intestinal dysbiosis and enhances barrier integrity [67,91,92,93]. 

Moreover, among the bacteria reduced by EGCG, Clostridia stand out as a group of Gram-positive bacilli that, when in excess, can lead to infection in the large intestine [28]. Clostridia produce significant compounds such as butyrate or butyric acid, along with other SCFAs generated from the fermentation of dietary fiber. [94]. Butyrate is an essential metabolite in the human colon as it is the preferred energy source for colonic epithelial cells. It contributes to maintaining intestinal barrier functions and has immunomodulatory and anti-inflammatory properties [95]. However, excessively high levels of this metabolite in the intestine promote intestinal permeability, which may allow the passage of toxic substances into the bloodstream, potentially leading to inflammation [94]. Thus, EGCG’s inhibitory effect on this bacterial group, as observed in rats and resulting in decreased levels of *Clostridium* spp., balancing their levels, is beneficial [72]. This is particularly relevant for *Clostridium perfringens*, as it significantly impacts disease symptomatology, including gastrointestinal issues. Both the bacterium itself and the gene that produces its toxin (CPB2) have been linked to gastrointestinal complications in ASD and are correlated with disease severity. Moreover, the isolated species from children with ASD show greater antibiotic resistance compared to healthy children [96]. Conversely, the increase in *Clostridium difficile* has a notably negative impact on ASD, and effective treatments against this bacterium using oral vancomycin have shown improvements in behavior and communication [97,98]. Therefore, the reduction of this strain mediated by EGCG could be beneficial for the clinical presentation of children affected by ASD.

EGCG treatment also leads to a decrease in enterobacteria, which are commonly present in the body but can cause infections when their growth is uncontrolled. Certain species have been linked to specific pathologies. In particular, *Salmonella typhi* is responsible for typhoid fever, *Shigella dysenteriae* is the causative agent of bacillary dysentery and causes infantile gastroenteritis, and *Yersinia pestis* causes plague [99]. 

Lastly, it is important to highlight that specific bacteria increased by EGCG, such as *Lactobacillus acidophilus*, *Bifidobacterium longum*, *Akkermansia muciniphila*, and *Prevotella ruminicola*, restore the balance of the intestinal microbiota when included to the diet, reducing oxidative stress in the intestine and the brain [100] (Table 1).

### 3.2. Outlook on the Anti-Inflammatory and Antioxidant Activity of EGCG in Autism: Neuroprotective Role

Immune system dysregulation, inflammation, and increased oxidative stress are significant factors in ASD [101], which is directly linked to dysbiosis. Comorbid inflammatory conditions associated with immune dysregulation closely contribute to the emergence and progression of clinical features in children with ASD [102,103,104]. Cytokines are small proteins regulating inflammation and neurological development. Increased levels of some of them, particularly, IL-4 o IL-10, have been seen in children with ASD [105]. Notable changes in interferon-α (IFN-α), interleukin-7 (IL-7), IFN-γ-inducible protein-10, and IL-8 are particularly relevant to the disorder’s pathogenesis [106].

IL-1β and IL-10 levels produced by blood monocytes, along with the IL-1β/IL-10 ratio and miRNA expression, are relevant to innate immunity [107,108]. All these alterations are closely associated with social behavioral impairments and cognitive development in children with ASD [109], which could be due to a brain dysfunction associated with the proper production of growth factors and neurogenesis [110]. In particular, the brain-derived neurotrophic factor (BDNF) plays important roles in the formation, branching, and connectivity of synaptic connections during development [111,112], as well as in synaptic plasticity, as it is involved in learning and memory [113,114].

Oxidative stress is directly linked to intestinal microbiota, particularly in children with ASD [115]. The fermentation of dietary fibers and resistant starch in the gut generates SCFAs [116], with propionate being associated with gastrointestinal issues and neuroinflammation in ASD [117]. Consequently, oxidative stress, closely tied to dysbiosis, plays a crucial role in the neuroinflammatory response and the development of ASD [118,119,120,121,122]. Children with autism exhibit differences in antioxidant capacity and oxidative stress-related metabolites compared to healthy children [123]. The imbalance in glutathione levels and a decreased storage capacity contribute to increased susceptibility to oxidative stress in autistic children [124]. Moreover, increased free radical production, influenced by amyloid precursor proteins (APP), is observed in various human disorders, including autism [125,126,127].

Polyphenols, particularly EGCG, play a role as antioxidants and promote neurogenesis and plasticity in a Down syndrome mouse model [128], showing potential in alleviating autistic symptoms. Other polyphenols, such as cyanidin-3-glucosidereduce intestinal inflammation in human intestinal cells, affect inflammatory cascades like nuclear factor kappa B (NF-kB), activator protein-1 (AP-1), and Janus kinase-signal transducer and activator of transcription (JAK/STAT) [129]. Furthermore, resveratrol in vitro modulates inflammatory markers, including nitric oxide (NO), tumor necrosis factor-alpha (TNF-α), ionized calcium-binding adapter molecule 1 (Iba1), prostaglandin E2 (PGE2), inducible nitric oxide synthase (iNOS), and cyclooxygenase-(COX-2) [130]. Moreover, a study on rat pups with autistic traits found that a polyphenol–probiotic complex reversed autistic behaviors and modulated biochemical changes in IL-6, TNF-α, BDNF, 5-HT, AchE, and the granular layer, which is supported by all this evidence [131].

Therefore, when analyzing the activity of various polyphenols in inflammation and oxidative stress, these molecules have demonstrated high efficacy in regulating APP levels [132]. Its ability to cross the BBB (based on the hBMEC model, which utilizes human-derived brain endothelial cells), plays a significant role in protecting cortical neurons against oxidative stress-induced cell death. Comparatively, EGCG demonstrated rapid BBB permeation, while cyanidin-3-glucoside (C3G) showed slower permeation, and quercetin did not cross the BBB. This makes EGCG particularly promising as a neuroprotector among various polyphenols [133]. In relation to ASD, IL-8 stands out among the cytokines involved. The administration of oral EGCG reduces its levels via the inhibition of intracellular Ca2+ levels and the activation of ERK1/2 and NF-kappaB pathways. This is because it has been seen in the colon that the treatment of TNF-α-stimulated HT29 cells (human colon carcinoma cell lines) with EGCG inhibits IL-8 production by regulating genes in inflammatory pathways, suggesting its potential for preventing or mitigating colonic disorders in autism [134].

Finally, it should be noted that elevated levels of reactive oxygen species (ROS), specifically hydrogen peroxide (H_2_ O_2_) and superoxide (O_2_^−^), are reported in the intestine of children with ASD as a cause of damage to the epithelial tissue [135,136,137], and EGCG negatively regulates the inflammatory response in inflamed intestinal epithelial cells, largely via a post-transcriptional regulatory mechanism [138].

### 3.3. Role of EGCG in the Metabolic Activity Associated with Dysbiosis in ASD

EGCG regulates the production of SCFAs [42], which are the metabolites involved in the bidirectional communication between the gut and the brain. Succinate and butyrate are particularly important and can be altered in individuals with autism due to microbial activity [139]. An imbalanced ratio of succinate production/consumption can cause intestinal disturbances and impact the gut–brain axis [140]. Cheng et al. determined via a review that altered succinate levels have been linked to disrupted calcium homeostasis and dysregulated metabolic functions in autistic individuals [41].

Regarding butyrate, it has been seen that children with ASD have lower levels of fecal butyrate, as well as a decrease in the abundance of taxa producing this metabolite [141]. Butyrate is particularly relevant in ASD as its production mainly derives from the gut microbiome. Butyrate displays potent anti-inflammatory activity that contributes to gut health, regulates intestinal homeostasis, and modulates the expression of neurotransmitter genes [142,143]. Furthermore, it positively modulates the impaired mitochondrial function in ASD, characterized by reduced activity of complex IV in the electron transport chain (ETC) [144], including improvements in oxidative phosphorylation and beta-oxidation. These observations have underscored the significant neuroprotective role of butyrate in children with ASD [145]. In fact, it has recently been seen that maternal treatment with butyrate in the BTBR mouse model of ASD rescues social and partially repetitive behavior deficits in the offspring [146]. Thus, proper butyrate production at adequate levels is crucial, and this depends on the correct development of the gut bacterial community. Oral EGCG delivery on the DSS-induced murine colitis model has been shown to increase both the abundance of *Akkermansia muciniphila* and its butyrate production [88], potentially reversing the adverse effects resulting from altered butyrate levels in ASD. On the other hand, among the altered fecal metabolite profiles in children with ASD (not present in neurotypical children), there is an increase in p-cresol, caprate, and aspartate and a reduction in GABA, nicotinate, glutamine, and thymine [147]. Among all these metabolites, p-cresol is a uremic toxin produced by certain strains of *Clostridium* spp., which has negative biological effects and appears to adversely affect the homeostasis of colonic epithelial cells in children with ASD. When present in excess, p-cresol induces DNA damage in vitro and negatively affects the integrity of colonic epithelial cells [148]. In fact, EGCG may have beneficial effects by reversing the activity of this metabolite. In a recent study, it has been seen that an EGCG-enriched diet reduces plasma and urinary concentrations of p-cresol in mice by suppressing the abundance of Firmicutes at the phylum level and Clostridia at the order level [149].

**Figure 1:**
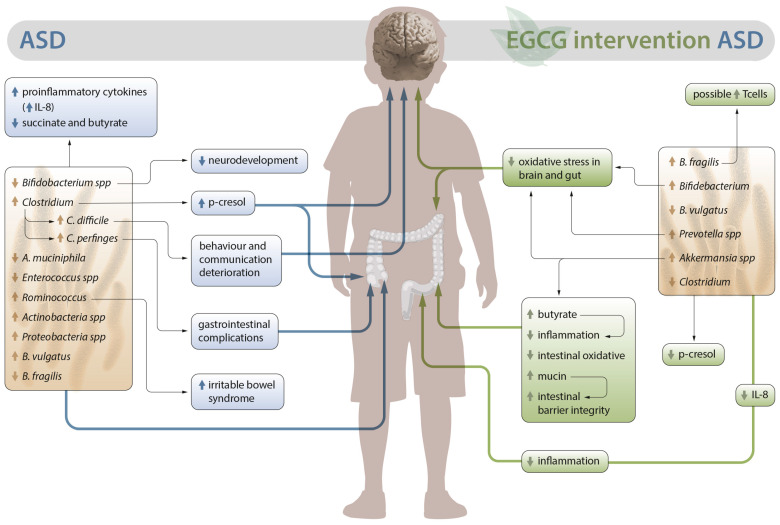
EGCG impact in patients with ASD. Alterations in the intestinal microbiota in ASD and its implications on the left; EGCG impact on both the intestinal microbiota and variables directly related to the microbiota such as metabolites, inflammation, and oxidative stress on the right. (↑) means increase and (↓) means decrease.

In conclusion, EGCG has an impact on both the intestinal microbiota and variables directly related to the microbiota such as metabolites, inflammation, and oxidative stress, which could provide benefits for patients with ASD (Figure 1).

**Figure 2:**
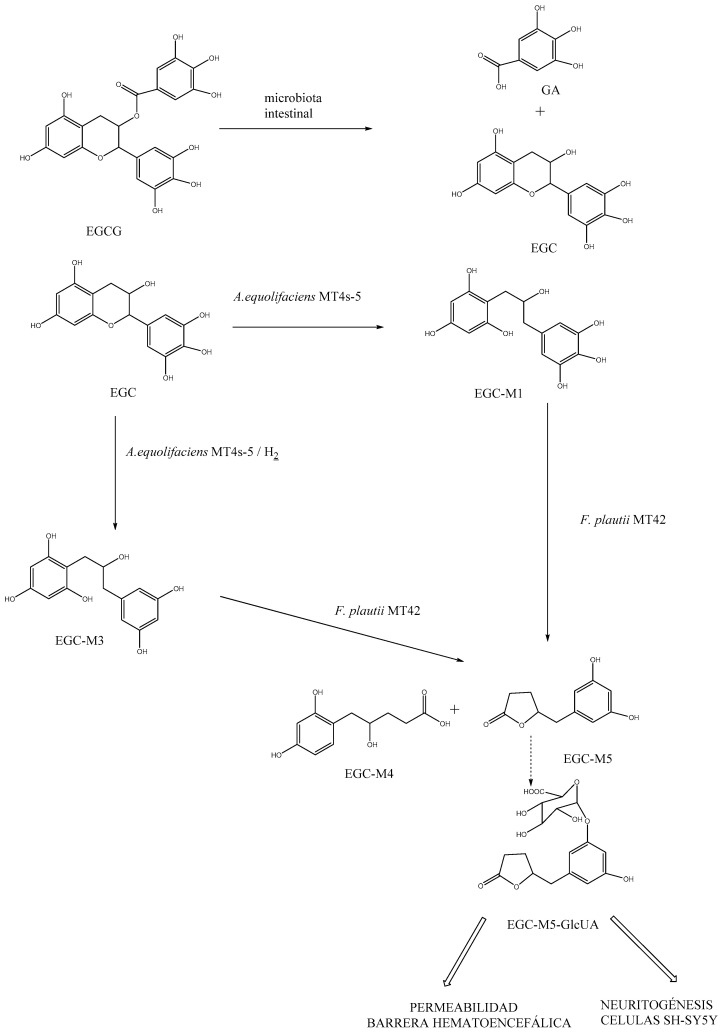
Mechanism of EGCG metabolite generation in the intestinal microbiota. Reciprocal interactions between EGCG and human gut microbiota in vitro [65].

Another noteworthy aspect is the impact of metabolites generated from the interaction between the polyphenol and the intestinal microbiota. Particularly, in rats, the metabolic pathway of EGCG leads to gallic acid (GA) and epigallocatechin (EGC) [150]. EGC is degraded via colonic bacteria to yield microbial ring-fission metabolites, specifically converting to 5-(3′,5′-dihydroxyphenyl)-γ-valerolactone (EGC-M5). Then, the bacteria Adlercreutzia equolifaciens MT4s-5 and Flavonifractor plautii MT42 are capable of degrading EGC. The bacterium Adlercreutzia equolifaciens MT4s-5 catalyzes the conversion of EGC to 1-(3,4,5-trihydroxyphenyl)-3-(2,4,6-trihydroxyphenyl)propan-2-ol (EGC-M1), and subsequently, Flavonifractor plautii MT42 converts the propan-2-ol to 5-(3,4,5-trihydroxyphenyl)-γ-valerolactone (EGC-M5) and 4-hydroxy-5-(3,4,5-trihydroxyphenyl)valeric acid (EGC-M4). Similarly, EGC-M5 is produced from 1-(3,5-dihydroxyphenyl)-3-(2,4,6-trihydroxyphenyl)propan-2-ol (EGC-M3), which is formed from EGC via Adlercreutzia equolifaciens MT4s-5 in the presence of hydrogen [120]. A large portion of the formed EGC-M5 is absorbed and undergoes glucuronidation in the intestinal mucosa and/or liver to form EGC-M5 glucuronide (EGC-M5-GlcUA), which is distributed to various tissues through the bloodstream and ultimately excreted in urine [151] (Figure 2).

The microbial ring-fission metabolites of EGCG are found in plasma in both free and conjugated forms [151], and in vitro studies have shown that they may reach the brain parenchyma through the BBB, promoting neuritogenesis and potentially exerting a relevant activity against the degenerative processes of neurodegenerative diseases [152].

Specifically, when assessing the penetration capacity of these metabolites through the BBB, it has been seen that GA has higher permeability than EGCG and EGC, possibly due to its smaller molecular size [153]. Moreover, comparing only the ring-fission metabolites derived from ECG, EGC-M5 demonstrated greater permeability than its conjugate EGC-M5-GlcUA [150]. Some of these ring-fission metabolites also exhibit anti-inflammatory activity [154]. In particular, EGC-M5 has been found to have immunomodulatory properties, as it enhances the activity of CD4+ T cells and the cytotoxic activity of natural killer cells in BALB/c mice [155]. Therefore, it seems evident that the ring-fission metabolites derived from the intestinal microbiota of catechins demonstrate a protective capacity against various diseases, including neurodegenerative disorders.

### 3.4. Liposomal EGCG

However, it is crucial to understand the metabolic process and bioavailability of green tea catechins and EGCG, particularly in assessing their biological activity and comprehending their beneficial effects on human health. EGCG presents significantly lower bioavailability compared to other components of catechins [156,157]. Therefore, when assessing the activity of EGCG and its metabolites, it is essential to emphasize that their applications are greatly limited due to their low solubility, bioavailability, and stability. The use of liposomal formulations may serve as an effective strategy for their administration in autism. Liposomal delivery aims to improve the poor stability of polyphenols against temperature, light, pH, and oxygen [28,29], as well as their low permeability across intestinal membranes, which results in only a small proportion of these compounds remaining available for absorption in the human body after ingestion [29,30]. Both stability and oral bioavailability are enhanced via liposomal encapsulation, as it provides protection against degradation when passing through the gastrointestinal tract [31]. Moreover, nanotechnology can promote the controlled release of the polyphenol and modulate the interaction between polyphenols and the microbiota, which also represents an intriguing approach [31], and it has been seen that EGCG has significantly improved stability when formulated as dual-drug-loaded PEGylated PLGA nanoparticles (EGCG/AA NPs). Following oral administration in mice, EGCG accumulated in all major organs, including the brain, leading to an increase in synapse formation and a reduction in neuroinflammation in Alzheimer’s disease [158].

These results seem to support the increase in EGCG activity when administered in a liposomal form, particularly enhancing its neuroprotective activity as seen in both in vivo and in vitro models of Parkinson’s disease [159]. Furthermore, concerning its metabolites and their administration using nanotechnology, Abbasalipour H. et al. (2022) studied the neuroprotective effects of gallic acid on oxidative stress-induced cognitive impairment and the expression of the Nrf2/Keap1 gene in an autism model. Gallic acid-loaded nanophytosomes (GNP) were administered, and the results revealed improvements in learning and memory deficits by reducing oxidative stress, enhancing antioxidant enzyme activity, and modulating the Keap1/Nrf2 gene expression [160].

### 3.5. Potential Adverse Effects of EGCG

Green tea and, to a lesser extent, black tea are not only the main sources of EGCG but also contain other molecules such as caffeine. It is noteworthy that caffeine consumption has been specifically linked to an increase in sleep disturbances, characteristic of this population [161], as well as the presence of maladaptive behavior in children with ASD [162]. Furthermore, concerning the microbiota in individuals vulnerable to *Clostridium difficile* infections, an increased risk in such infections has been associated with tea consumption, as it disrupts the normal microbiome and promotes the overgrowth of facultative pathogens [163]. Tea consumption has also been identified as a risk factor for gastroesophageal reflux disease (GERD) [164], which is relevant considering that GERD is one of the gastrointestinal symptoms commonly described as ASD [165]. Moreover, excessive consumption of EGCG has been found to be hepatotoxic [166,167,168].

Despite these considerations, tea has been consumed worldwide for many centuries, and the intake of its main catechin, EGCG, has been shown to be particularly safe even at doses up to 800 mg/day [169]. Thus, consuming tea at appropriate doses presents significant health benefits and is highly safe in terms of potential adverse effects or contraindications [170].

## 4. Conclusions

ASD is a complex disorder characterized by inflammatory processes, high oxidative stress, and gastrointestinal disturbances. These issues may be attributed to changes in the intestinal microbiome, resulting in microbial dysbiosis in children with ASD. Polyphenols have demonstrated sufficient antioxidant and anti-inflammatory capacity to counteract ASD symptoms. Their role in dysbiosis has also been studied, revealing their ability to regulate and improve the percentages of beneficial microbial species, thereby enhancing the pathology of ASD and promoting gut–brain axis function, consequently improving brain function. In this regard, EGCG appears particularly effective in improving microbial species and increasing the production of metabolites involved in maintaining epithelial integrity. It also demonstrates effectiveness in reducing inflammation directly linked to ASD by influencing specific pro-inflammatory cytokine levels and reducing oxidative stress. Furthermore, it can modulate the altered metabolic production in ASD by decreasing toxic metabolites such as p-cresol. All of these factors have an impact on brain function, which could provide significant benefits for children with ASD. However, it should be noted that these polyphenols have limitations in terms of intestinal assimilation and bioavailability. Hence, when considering the advantages of nanotechnology in overcoming these limitations, the administration of EGCG in liposomal form could potentially be more effective. Therefore, a future line of research could be to determine if the efficacy of liposomal ECGC is improved when administered as a complementary treatment for ASD.

Consequently, this review identifies EGCG liposomes as a potential adjunctive therapy for children with ASD, aiming to improve their quality of life and alleviate their symptoms.

## Figures and Tables

**Table 1:** Main results related to EGCG effects on microbiota populations.

Study	Study Type	Population/Sample Size	Intervention	Results	Ref.
Ankolekar C et al., 2011	Analytical Longitudinal Experimental	Cultures of *Helicobacter pylori* Lactic acid bacteria strains such as *B. longum*, *L. acidophilus*, and *L. plantarum*	They were exposed to tea preparations with rest times of 2 and 5 min	The 4 types of tea inhibited *H. pylori*	[47]
Shin JS et al., 2007	Analytical Cross-sectional Experimental	Cultures of bacteria like *S. aureus* and *E. coli*	The antibacterial activities of the main phenolic components of Camellia sinensis	(-)-epicatechin is more toxic against *S. aureus* and *E. coli*	[48]
Nakayama M et al., 2012	Analytical Longitudinal Experimental	Different cultures: *E. coli* O157:H7 producing Stx 1 and Stx 2 *S. aureus* NBRC 13276	Green tea extract concentration was 0.0625 mg/mL for *S. aureus* and 1.0 mg/mL for *E. coli* O157:H7.	Green tea extract (GTE) inhibits the absorption and secretion of substrates and inhibits enzyme activity in both species	[49]
Bancirova M et al., 2010	Analytical Cross-sectional Experimental	Cultures of standard strains of *Enterococcus faecalis* 4224, *S. aureus* 3953 and 4223, *P. aeruginosa* 3955 and *E. coli* 3954 and 3988	Tea infusion (2 g/100 mL)	*E. faecalis* was the most resistant bacterial strain and *P. aeruginosa* was the least resistant bacterial strain. But the predominant antimicrobial activity of non-fermented tea infusions was not confirmed.	[52]
Liu Z et al., 2020	Analytical Cross-sectional Experimental	Cultures of fecal material from four human volunteers	Treatment with catechin concentration of 0.1 mmol/L during 48 h.	EGCG increases the beneficial bacteria *Bacteroides*, *Christensenellaceae*, and *Bifidobacterium.* And inhibited the pathogenic bacteria *Fusobacterium varium*, *Bilophila*, and *Enterobacteriaceae*.	[65]
Trovò L et al., 2020	Analytical Longitudinal Experimental	In vivo study with Cdkl5-KO mice N = 5–6	Primary hippocampal neurons were treated daily with EGCG [1 μM, 0.5 μM, 1 μM, and 3 μM] or harmine	Treatment with EGCG efficiently restores defects in the dendritic and synaptic development of Cdkl5-KO hippocampal neurons.	[57]
Kumaravel P et al., 2017	Analytical Longitudinal Experimental	In vivo study with Wistar rats N = 6 per group	Rats were treated with EGCG in doses of 1, 2, and 5 mg/kg body weight via oral administration.	EGCG ameliorates and reverses autistic attributes possibly due to its neuroprotective activity.	[58]
Ushiroda C et al., 2019	Analytical Longitudinal Experimental	In vivo study with male C57BL/6 N mice	EGCG at a concentration of 0.32% for 8 weeks	EGCG increases the abundance *of Adlercreutzia*, *Akkermansia*, and *Allobaculum* and a significantly lower abundance of *Desulfovibrionaceae*	[67]
Qu Y et al., 2022	Analytical Longitudinal Experimental	In vivo study with female C57BL/6 mice (8–13 weeks old)	EGCG [45 mg/kg] was administered for 8 weeks. The mice were then subjected to behavioral and gut microbiota analysis	EGCG effectively increased *Prevotella* and decreased *Bifidobacteriales* but had no effect on *Alloprevotella* or *Lactobacillaceae*	[68]
Unno T et al., 2014	Analytical Longitudinal Experimental	In vivo study with four-week-old male Wistar rats N= 7 per group	Rats were fed an assigned diet of either a control diet, a 0.3% (*w*/*w*) EGCG diet, or a 0.6% (*w*/*w*) EGCG diet for 4 weeks	Dietary EGCG affects the growth of certain species of gut microbiota which could be responsible for regulating energy metabolism in the body	[72]
Wu Z et al., 2021	Analytical Longitudinal Experimental	In vivo study with seven- to eight-week-old specifc pathogen-free (SPF) female C57BL/6J mice N = 8 per group	Modulated the gut microbial community of mice by the EGCG [50 mg/kg] pre-supplementation	Oral EGCG suppressed DSS-induced oxidative stress in the intestinal mucosa, improved the barrier function, and regulated the composition and SCFAs production of gut microbiota	[88]
Wu Z et al., 2020	Analytical Longitudinal Experimental	In vivo study with seven- to eight-week-old specific pathogen-free (SPF) female C57L/6J mice	Tested the effect of oral or rectal administration of EGCG [50 mg/kg]	EGCG enriched *Akkermansia*, *Faecalibaculum*, and *Bifidobacterium* and enhanced acetate, propionate, and butyrate production.	[89]
Sheng L et al., 2018	Analytical Longitudinal Experimental	In vivo study with specific pathogen-free male C57BL/6 wild-type (WT) mice N = 16	Mice received vehicle (PBS) or EGCG daily [100 mg/d per gram body weight, orally] for 2 mo.	Increased *Verrucomicrobiaceae* and *Akkermansia muciniphila.*	[92]
Liu X et al., 2020	Analytical Longitudinal Experimental	In vivo study with C57BL/6J male mice N = 8	C57BL/6J mice fed with HFD were administrated with 210 mg/kg EGCG for 12 weeks.	Enriched the *Verrucomicrobia* and decreased the *Firmicutes* and *Saccharibacteria.*	[93]

## Data Availability

Not applicable due to data privacy.
